# Cyclosporin A, but not tacrolimus, negatively affects the hepatic extraction fraction of hepatobiliary scintigraphy in liver transplant recipients

**DOI:** 10.1186/s13550-014-0073-z

**Published:** 2014-12-17

**Authors:** Anastasios Kaxiras, Shinji Yamamoto, Gunnar Söderdahl, Annika Wernerson, Rimma Axelsson, Bo-Göran Ericzon

**Affiliations:** Division of Transplantation Surgery and CLINTEC, Karolinska University Hospital, Huddinge, and Karolinska Institutet, Stockholm, Sweden; Clinical Pathology/Cytology and CLINTEC, Karolinska University Hospital, Huddinge and Karolinska Institutet, Stockholm, Sweden; Division of Radiology and CLINTEC, Karolinska University Hospital, Huddinge and Karolinska Institutet, Stockholm, Sweden

**Keywords:** Hepatobiliary scintigraphy, Hepatic extraction rate, Liver transplantation, Cyclosporin A, Tacrolimus, Hepatitis C

## Abstract

**Background:**

Hepatobiliary scintigraphy using ^99m^Tc-mebrofenin has been used as an investigation to study liver function after liver transplantation (LTx). Hepatic extraction fraction (HEF) is a measurement of the hepatic extraction efficiency and hepatic extraction rate. With the purpose of evaluating a possible diverging effect of cyclosporin A (CSA) and tacrolimus (TAC) on the HEF, we compared the HEF with biochemical and histological parameters in LTx patients receiving either CSA or TAC.

**Methods:**

Thirty-nine adult patients who underwent LTx due to hepatitis C virus (HCV) cirrhosis were evaluated. All patients underwent a 3-month and 1-year follow-up that included hepatobiliary scintigraphy and biochemistry tests. Liver biopsy was performed at 1 year. These clinical parameters were compared between the two groups, TAC (*n* = 15) and CSA (*n* = 24).

**Results:**

The average HEF was significantly lower in the CSA group compared to the TAC group both at 3 months and 1 year after LTx. The liver biochemistry tests, average donor and recipient age, average cold ischemia time (CIT), and a clearance were comparable in the two groups. The TAC group had more inflammation than the CSA group. Moreover, three patients who converted from CSA to TAC increased their HEF values.

**Conclusions:**

CSA-treated patients presented a lower HEF value on hepatobiliary scintigraphy in spite of comparable liver function by traditional measurements indicating a decrease on HEF values by CSA.

## Background

Liver transplantation (LTx) is an established treatment for patients with end-stage liver disease, and LTx has been performed in Sweden since 1984 [1]. Liver cirrhosis caused by hepatitis C virus (HCV) is a common indication for LTx [2]. Approximately one-fifth of transplant recipients at our institute had liver cirrhosis caused by HCV [3]. Recurrence of HCV infection can occur as early as 4 weeks after liver transplantation [4]; this may develop into severe hepatitis requiring anti-viral treatment within the first 6 months after LTx [5-7]. The recurrence of HCV infection is usually diagnosed by blood biochemistry tests, HCV-RNA test, and liver graft biopsy. Other methods are also used for follow-up and to evaluate liver graft function after LTx. These include radiological examinations, transient elastography (fibroscan), and hepatobiliary scintigraphy. Hepatobiliary scintigraphy has emerged as one of the valuable methods to assess graft function [8-14]. The most common agent used for nuclear imaging with scintigraphy worldwide and by us is ^99m^Tc-mebrofenin (2,4,6-trimethyl-3 bromo iminodiacetic acid) [14-16]. Hepatobiliary scintigraphy is a noninvasive method and allows for detection of post-operative structural complications such as biliary obstructions/dilatations and biliary leakage. In addition, it can provide information concerning functional components of the liver graft, such as the rate of uptake, perfusion, and excretion of a tracer [9]. One important parameter of biliary scintigraphy that is of great value in the evaluation of transplanted liver grafts is the hepatic extraction fraction (HEF) [14]. HEF is a measurement of the hepatic extraction efficiency and hepatic extraction rate [14-16]. We have previously reported a significant correlation between early post-LTx HEF values and biochemical parameters [14].

A high level of serum bilirubin in the blood is known to interact with the uptake and/or excretion of scintigraphic agents in the liver, thereby affecting the scintigraphy results [14]. A previous study performed in an animal model showed that cyclosporin A (CSA) possibly impaired the ^99m^Tc-mebrofenin excretion [17]. It might indicate that CSA affects the rate of HEF negatively. However, this negative effect has not been proved in scintigraphy with humans so far. In this retrospective study, we analyzed the results of hepatobiliary scintigraphy performed at the 3-month and 1-year post-transplantation check-up in patients transplanted as a result of HCV cirrhosis. We then compared the scintigraphic results to the traditional biochemical and histological parameters between the two groups of patients, receiving either CSA or tacrolimus (TAC) with the purpose of evaluating a possible diverging effect of these drugs on the HEF.

## Methods

### Patients

Thirty-nine adult patients who underwent LTx due to a single indication, i.e., HCV cirrhosis at the Karolinska University Hospital between March 2007 and May 2011, were included in the study. During this time period, our center performed routine hepatobiliary scintigraphy at 3 and 12 months after LTx. Thirty-eight patients received a whole-liver graft (*n* = 38) and one received a right lobe graft from a deceased donor. The patients were divided into two groups, those that received TAC from the first day after transplantation (TAC group, *n* = 15) and those that received CSA (CSA group, *n* = 24). Several clinical parameters at the time of transplantation were also analyzed, including donor and recipient age, recipient gender, malignancy and the cold ischemia time (CIT). Characteristics of the patients in both groups are shown in Table 1. The study was approved by the review board of the ethical committee at Karolinska Institutet, Sweden.

**Table 1:** **Characteristics of 39 HCV positive patients who underwent LTx**

	**Total (** ***n*** **= 39)**	**Cyclosporine A (** ***n*** **= 24)**	**Tacrolimus (** ***n*** **= 15)**	***p*** **value**
Recipient age (years) (range, average ± S.D.)	42 to 67	43 to 67	42 to 66	0.62
	56.1 ± 6.6	55.7 ± 6.8	56.8 ± 6.3	
Gender (male/female)	25/14	14/10	11/4	0.90
HCC (%)	17 (43.6%)	12 (50%)	5 (33.3%)	0.31
Donor age (years) (range, average ± SD)	16 to 74 (54.5 ± 13.8)	16 to 74 (53.3 ± 17.0)	43 to 64 (56.3 ± 6.4)	
Cold ischemic time (minutes, range, average ± SD)	240 to 775 (501.3 ± 121.9)	350 to 751 (513.3 ± 111.9)	240 to 775 (482.1 ± 138.3)	
Post-operative complication with bile duct stricture/bile leakage	4 (10.3%)	3 (12.5%)	1 (6.7%)	0.56

LTx, liver transplantation; SD, standard deviation; HCC, hepatocellular carcinoma.

### Immunosuppression

During the study period, the selection of CSA or TAC in patients with HCV cirrhosis was based on the operating surgeons' own choice and no special criteria were used (CSA; *n* = 14, TAC; *n* = 15). However, after November 2010, only CSA was used in these patients (CSA; *n* = 10). All patients in the TAC group received a double-regime immunosuppression protocol without induction therapy, i.e., TAC + cortisone. The patients in the CSA group received a triple immunosuppression regime without induction therapy, i.e., CSA + Azathioprine (AZA) + cortisone. The initial dose was 0.1 mg/kg/day for the TAC group and 10 mg/kg/day for the CSA group, both divided in two doses a day. The target trough level in the first three post-operative months was 8 to 10 ng/ml for the TAC group and 200 to 300 ng/ml for the CSA group. Beyond the 3 months, the target level was reduced to 4 to 6 ng/ml for the TAC group and 150 to 200 ng/ml for the CSA group. In the CSA group, CSA was converted to TAC treatment in three patients before the 1-year follow-up scintigraphy was performed. In the TAC group, TAC was converted to everolimus in one patient at the time of the 1-year follow-up. According to our protocol, the initial dosage of AZA was 3 mg/kg at a maximum dose of 150 mg/day, reduced to 1 to 2 mg/kg after the 3-month follow-up and subsequently withdrawn after the 1-year follow-up. However, nine patients in the CSA group had the AZA removed earlier than 1 year after LTx because of side effects, mainly anemia and/or leucopenia. The time of drug removal varied from 2 weeks to 9 months after LTx (median, 3.0 months).

### Follow-up of the patients

All patients underwent a 3-month follow-up that included hepatobiliary scintigraphy and blood biochemistry tests: s-bilirubin, alanine aminotransferase (ALAT), aspartate aminotransferase (ASAT), alkaline phosphatase (ALP), and gamma-glutamyl transpeptidase (GT). Hepatobiliary scintigraphy and the same blood biochemistry tests were repeated at the 1-year follow-up; in addition creatinine and iohexol clearance and a liver biopsy were performed at 1 year.

### Hepatobiliary scintigraphy

#### Procedure

**Figure 1:**
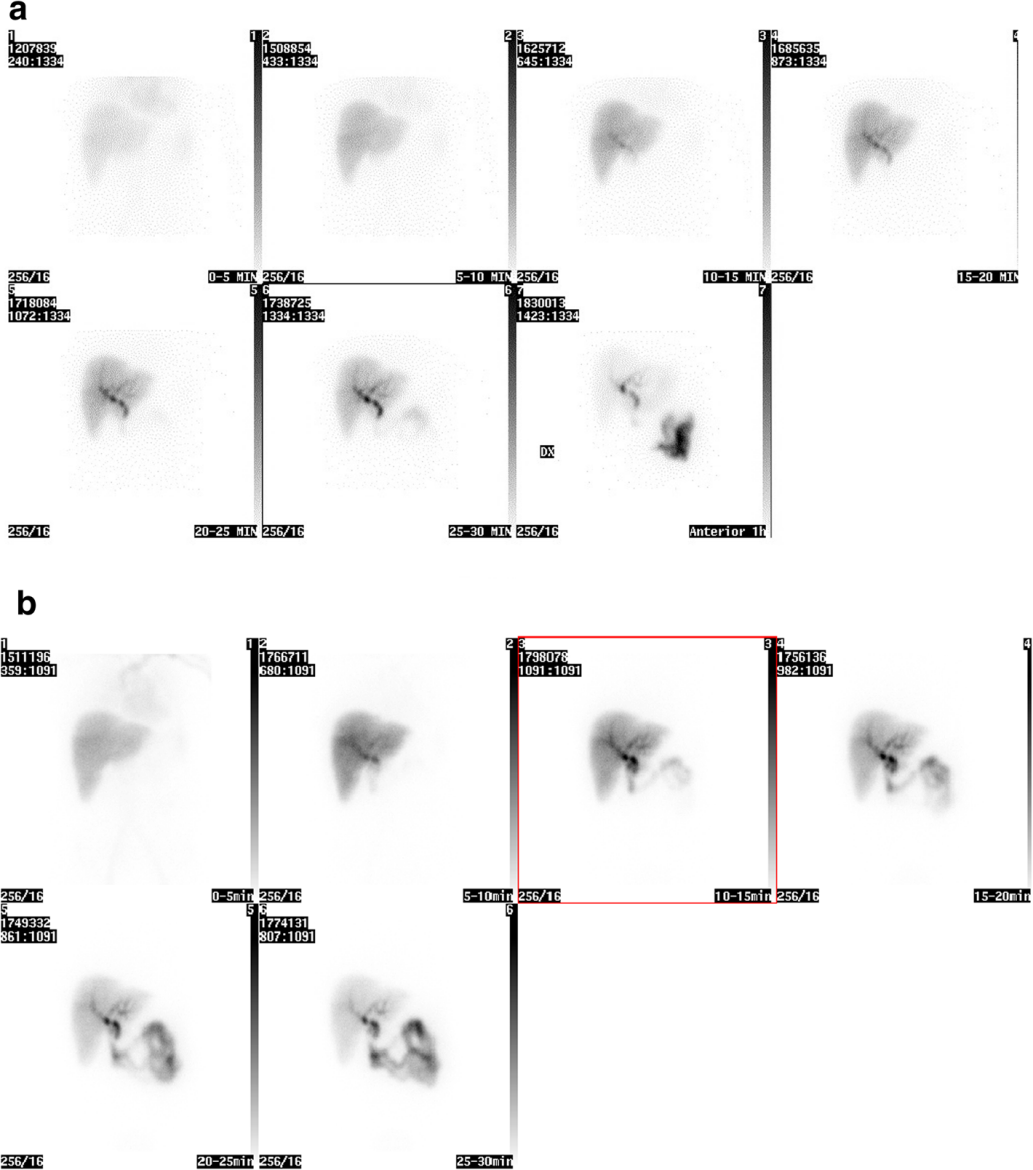
**Hepatobiliary scintigraphy.** A 53-year old female patient who underwent liver transplantation (LTx) due to hepatitis C virus cirrhosis (case 2 in Table 4). Cyclosporin A was converted to tacrolimus treatment due to peripheral neuropathy 5 months after LTx. **(a)** A hepatobiliary scintigraphy with ^99m^Tc-Mebrofenin performed 3 months after LTx. Dynamic study with recording time of 30 min in anterior projection. Images show a homogeneous accumulation of ^99m^Tc-Mebrofenin in the transplanted liver and excretion to the small bowel at 15 to 20 min after injection. **(b)** A hepatobiliary scintigraphy with ^99m^Tc-Mebrofenin in the same patient performed 1 year after LTx. Visually homogeneous accumulation of ^99m^Tc-Mebrofenin in the transplanted liver and excretion to the small bowel in 10 to 15 min after injection.

Hepatobiliary scintigraphy was performed on a large field-of-view gamma camera (Diacam; Siemens, Munich, Germany) at 3 months and 12 months post-operatively. A dynamic acquisition was started directly after a bolus injection of 200 MBq ^99m^Tc mebrofenin, which is rapidly taken up by hepatocytes from circulating blood and extracted into the biliary tract without conjugation (Figure 1a,b). Data were acquired at a rate of one frame per 10 s over 30 min using a 256 × 256 matrix. The field of view included the heart, the liver, and the upper abdomen. A 5-min anterior view was acquired 1 h after injection.

#### Evaluation

The HEF was calculated using a restricted matrix algorithm deconvolution technique [17]. A region of interest (ROI) was drawn over the left ventricle of the heart, excluding the aorta and scatter from the liver. A second ROI was drawn over the entire liver parenchyma, excluding the main bile duct from the ROI in order to eliminate the risk of overlapping of excreted radioactivity from bile duct and the true radioactivity of the liver parenchyma (gallbladder was removed from the liver graft during operation).

**Figure 2:**
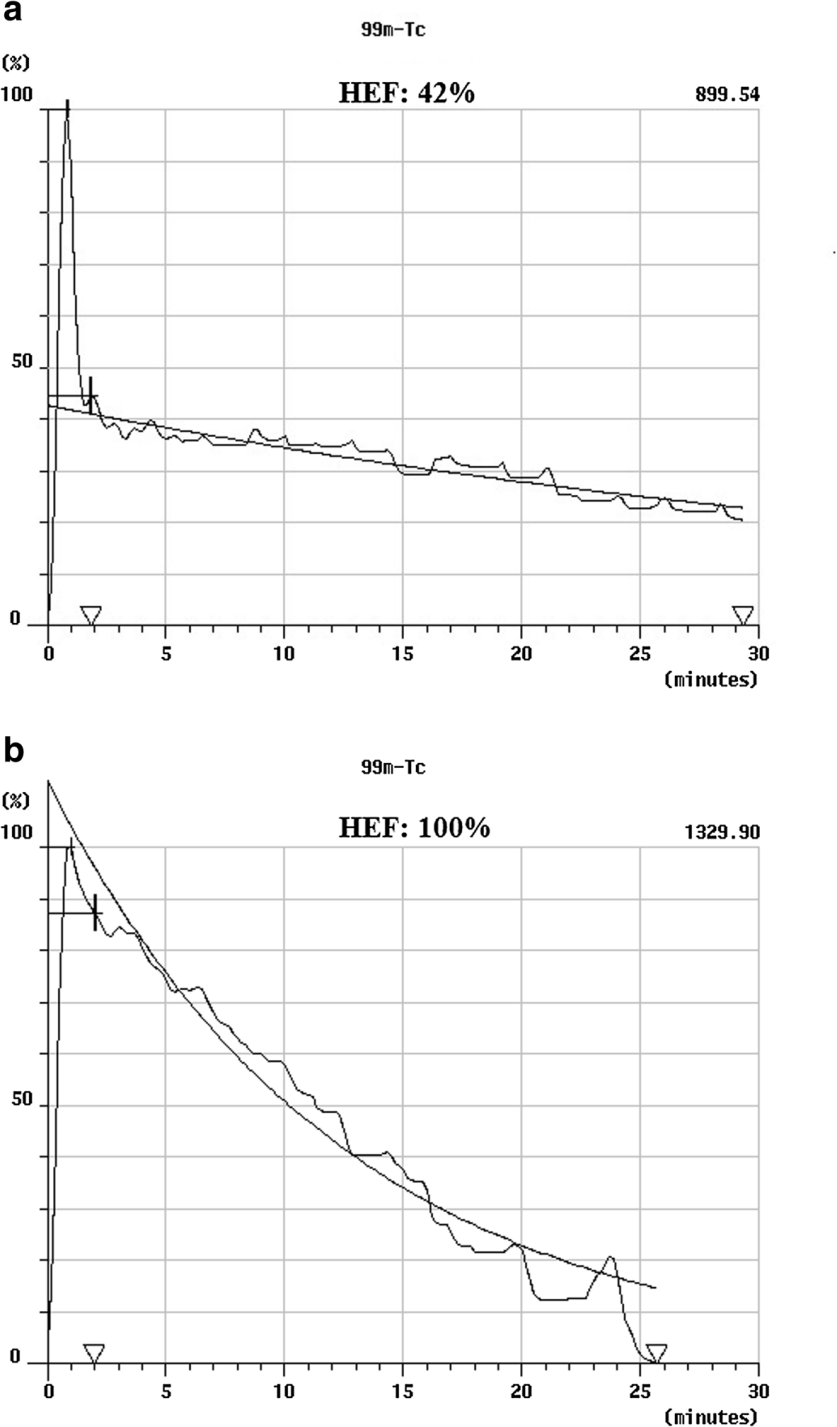
**Hepatic extraction fraction.** A 53-year old female patient who underwent liver transplantation (LTx) due to hepatitis C virus cirrhosis (case 2 in Table 4). Cyclosporin A was converted to tacrolimus treatment due to peripheral neuropathy 5 months after LTx. **(a)** Calculation of hepatic extraction fraction (HEF) in the same patient at 3 months imaging. HEF is calculated by the ratio of the initial hepatocyte uptake divided by the peak vascular uptake (42%). **(b)** Calculation of HEF in the same patient 1 year after LTx. HEF increased from 42% to 100%.

Time-activity curves were generated from these regions, and the heart curve was used as the input function in the deconvolution analysis. The deconvoluted liver curve thus represents a hypothetical true liver response following a direct bolus injection into the hepatic artery. An exponential curve was fitted by the least-squares method to the deconvoluted liver curve. The intercept of the fitted curve is proportional to the hepatocyte function, while the maximum of the deconvoluted liver curve represents the total input to the liver. HEF is calculated as: HEF = intercept exponential-fit liver response curve/max. data value liver response curve (Figure 2a,b).

### Liver biopsies

All liver biopsies were performed under the guidance of ultrasound, and all were analyzed by one experienced pathologist. The findings recorded were inflammation, fibrosis, steatosis, and the histological evidence of HCV recurrence. The grading system for the evaluation of the degree of inflammation/fibrosis/steatosis is as follows: 0: none, 1: light, 2: moderate, and 3: severe. HCV recurrence was diagnosed by the presence of focally intense lymphocytic infiltrates in portal areas, focal infiltration of a single interlobular bile duct without duct loss, a variable degree of piecemeal necrosis, and the absence of severe portal/hepatic venular endotheliitis [5].

### Statistical analysis

The STATISTICA statistical program (Statsoft inc., Tulsa, OK, USA) was used for the statistical analysis in this study. The chi-square test was performed for categorical variables and Student *t* test was performed for continuous variables. A *p* values < 0.05 was considered as statistically significant.

## Results

### Characteristics of CSA and TAC groups

In Table 1, the characteristics of both patient groups are shown. There was no statistical difference of recipient, donor and graft parameters, and post-operative complication between the CSA group and the TAC group. Three patients in the CSA group and two patients in the TAC group received intravenous bolus steroid therapy for biopsy-verified acute rejection within 3 months after LTx. There was no statistical difference of the occurrence of acute rejection between the two groups.

### Scintigraphic and biochemical parameters at a 3-month follow-up

The time intervals between LTx and 3-month follow-up hepatobiliary scintigraphy were 2.7 to 3.5, 3.1 ± 0.2 months (range, average ± standard deviation (SD)). There was a significant difference of the average of HEF values at the 3-month hepatobiliary scintigraphy; 11 to 90, 35.8 ± 21.2% (range, average ± S.D.) in the CSA group and 46 to 100, 87.3 ± 14.8% in the TAC group (*p* < 0.0001). Average of serum bilirubin was normal in both groups but significantly higher in the CSA group. There was a significant difference in the value of bilirubin between the two groups, (18.1 ± 13.5 vs 8.9 ± 8.4 μmol/l, respectively; *p* = 0.02). ALAT, ASAT, ALP, and GT were similar between the two groups (Table 2).

**Table 2:** **Results of scintigraphic and biochemical parameters in CSA and TAC groups at 3**-**month control**

**Parameters (range, average ± SD)**	**Total (** ***n*** **= 39)**	**CSA at 3 months (** ***n*** **= 24)**	**TAC at 3 months (** ***n*** **= 15)**	***p*** **value**
HEF (%)	11 to 100	11 to 90	46 to 100	<0.0001
	55.6 ± 31.6	35.8 ± 21.2	87.3 ± 14.8	
Bilirubin (μmol/l)	2.0 to 59.0	5.0 to 59.0	2.0 to 38.0	0.02
Ref: <26	14.6 ± 12.6	18.1 ± 13.5	8.9 ± 8.4	
ALAT (μkat/l)	0.1 to 1.6	0.1 to 1.6	0.2 to 1.0	0.57
Ref: <0.76	0.6 ± 0.3	0.6 ± 0.4	0.5 ± 0.2	
ASAT (μkat/l)	0.2 to 2.5	0.2 to 2.5	0.2 to 1.2	0.33
Ref: <0.61	0.6 ± 0.4	0.7 ± 0.5	0.5 ± 0.2	
ALP (μkat/l)	0.8 to 18.1	0.8 to 5.8	0.8 to 18.1	0.16
Ref: <1.9	2.1 ± 2.9	1.6 ± 1.2	3.0 ± 4.4	
GT (μkat/l)	0.3 to 22.7	0.3 to 8.8	0.4 to 22.7	0.16
Ref: <1.3	2.8 ± 4.6	2.0 ± 2.3	4.1 ± 6.7	

CSA, cyclosporine A; TAC, tacrolimus; ALAT, alanine aminotransferase; ASAT, aspartate aminotransferase; ALP, alkaline phosphatase; GT, gamma-glutamyl transpeptidase.

### Scintigraphic and biochemical parameters 1-year after LTx

The time intervals between LTx and 1-year follow-up hepatobiliary scintigraphy were 11.4 to 12.9, 12.1 ± 0.4 months (range, average ± SD). The average of HEF in the CSA group was significantly lower at 1 year after LTx than in patients treated with TAC (17 to 79, 44.7 ± 18.4% vs 37 to 100, 85.2 ± 23.6% (range, average ± SD); *p* < 0001). The average HEF in CSA treated patients who were on treatment with AZA at 1 year was 44.9 ± 19.4 compared to 53.3 ± 28.0 in patients who discontinued AZA (*p* = 0.39). There was no statistical difference in the liver and renal function by blood biochemistry evaluation between the CSA and TAC groups (Table 3).

**Table 3:** **Results of scintigraphic and biochemical parameters in CSA and TAC groups at 1**-**year control**

**Parameters (range, average ± SD)**	**Total (** ***n*** **= 38)**	**CSA at 1 year (** ***n*** **= 21)**	**TAC at 1 year (** ***n*** **= 17)**	***p*** **value**
HEF (%)	17 to 100	17 to 79	37 to 100	<0.0001
	62.8 ± 29.0	44.7 ± 18.4	85.2 ± 23.6	
Bilirubin (μmol/l)	3.0 to 146.0	4.0 to 146.0	3.0 to 142.0	0.88
	18.5 ± 30.4	17.8 ± 29.9	19.4 ± 32.0	
ALAT (μkat/l)	0.2 to 3.9	0.2 to 3.0	0.2 to 3.9	0.13
	1.1 ± 1.0	0.8 ± 0.7	1.3 ± 1.2	
ASAT (μkat/l)	0.3 to 3.0	0.3 to 2.4	0.3 to 3.0	0.38
	0.9 ± 0.7	0.8 ± 0.5	1.0 ± 0.9	
ALP (μkat/l)	0.7 to 5.8	0.7 to 5.8	0.8 to 4.8	0.44
	1.8 ± 1.0	1.9 ± 1.4	1.7 ± 1.0	
GT (μkat/l)	0.2 to 26.2	0.4 to 8.1	0.2 to 26.2	0.12
	3.2 ± 5.1	2.1 ± 2.3	4.6 ± 7.0	
Creatinine, (μmol/l)	60.0 to 273.0	60 to 273.0	63 to 177.0	0.23
	117.1 ± 44.6	124.9 ± 53.6	107.5 ± 28.7	
Iohexol clearance, (ml/min/1.7 m^2^)	6.4 to 91.0	18.0 to 78.0	6.4 to 91	0.33
	51.3 ± 17.4	48.8 ± 16.0	54.4 ± 19.0	

### HEF of patients who converted from CSA to TAC

Table 4 shows the HEF and serum bilirubin values at 3 months and 1 year after LTx in three patients who converted from CSA to TAC. Conversion was done between 3.7 to 6.3 months post-operatively. One patient (case 1) had normal bilirubin at the time of the two follow-ups and HEF increased from 27% to 90%. The second patient (case 2) who had slightly increased bilirubin at 3 months but a normal value at 1 year had HEF increased from 42% to 100% after conversion. Hepatobiliary scintigraphy and HEF of this case 3 months and 1 year after LTx are shown in Figures 1 and 2. The third patient (case 3) who had post-operative complication with bile duct stricture increased HEF from 13% to 26% in spite of a significant increase in serum bilirubin from 41 to 142 μmol/l.

**Table 4:** **Results of scintigraphic parameters in patients who converted from CSA to TAC at 1**-**year control**

	**Case 1**	**Case 2**	**Case 3**
Time of converting after LTx	3.7 months	5.0 months	6.3 months
Reason for converting	Side effect (hair growth)	Side effect (peripheral neuropathy)	Side effect (altered mental status)
3-month HEF (%)	27	42	13
Bilirubin, 3-month follow-up (μmol/l)	13	29	41
1-year HEF (%)	90	100	26
Bilirubin, 1-year follow-up (μmol/l)	10	8	142

### HEF inpatients with post-surgical complications

Post-surgical complications were evaluated in the patients. No re-operation was performed during the follow-up period. Four patients (2 in CSA group, 1 in TAC group and 1 in CSA converted to TAC at 6.3 months after LTx) presented with bile duct stricture (*n* = 3) and bile leakage (*n* = 1). Bile duct stricture and leakage was treated by dilatation of the stricture and bile duct stent placement with endoscopic retrograde cholangiopancreatography (ERCP) and percutaneous transhepatic cholangio drainage (PTCD). Table 5 shows the HEF and serum bilirubin values in these patients. Case 1 in the CSA group had normalized bilirubin value at 1 year; however, the HEF decreased from 46% to 37%. Case 2 in the TAC group had bile duct complications with high bilirubin values at both 3 months and 1 year after LTx even after the intervention and had low HEF values; 14% and 17%, respectively. Case 4 in the CSA group, who had complication of bile leakage after the removal of the T-tube at 1 month after LTx, had normal bilirubin values at 3 months and 1 year after LTx; however, the HEF values were low; 29% and 41%, respectively.

**Table 5:** **Results of scintigraphic parameters in patients who had bile duct complications**

**Case and immunosuppression**	**Case 1 CSA**	**Case 2 TAC**	**Case 3 CSA to TAC**	**Case 4 CSA**
Complications, time of the onset of treatment after LTx	Bile duct stricture, 4 months	Bile duct stricture, 1.5 months	Bile duct stricture, 5 months	Bile leakage after the removal of T-tube, 1 month
Treatment	Dilatation and bile duct stent placement by ERCP, PTCD	Dilatation and bile duct stent placement by ERCP, PTCD	Dilatation and bile duct stent placement by ERCP	Bile duct stent placement by ERCP, PTCD
3-month HEF (%)	46	14	13	29
Bilirubin, 3-month follow-up (μmol/l)	38	59	41	16
1-year HEF (%)	37	17	26	41
Bilirubin, 1-year follow-up (μmol/l)	8	146	142	18

LTx, liver transplantation; ERCP, endoscopic retrograde cholangiopancreatography; PTCD, percutaneous transhepatic cholangio drainage.

### Liver biopsy at the 1-year follow-up

The results of the liver biopsy taken 1 year after LTx are shown in Table 6. There was no significant difference in degrees of fibrosis and steatosis between the CSA and TAC groups; however, there was a significantly higher degree of inflammation in the TAC group compared to the CSA group (*p* = 0.005). The rate of the histopathologically confirmed recurrence of hepatitis C was 57.1% (12/21) in the CSA group and 50.0% (7/14) in the TAC group (*p* = 0.68).

**Table 6:** **Results of pathological parameters with CSA and TAC at 1-year control**

	**CSA (** ***n*** **= 21)**	**TAC (** ***n*** **= 14)**	***p*** **value**
Inflammation			
Low grade (0: none/1: light)	16 (2/14)	4 (1/5)	0.005
High grade (2: moderate/3: severe)	5 (6/0)	10 (8/2)	
Fibrosis			
Low grade (0: none/1: light)	12 (2/10)	6 (2/4)	0.40
High grade (2: moderate/3: severe)	9 (7/2)	8 (4/4)	
Steatosis			
Low grade (0: none/1: light)	13 (2/11)	11 (5/6)	0.30
High grade (2: moderate/3: severe)	8 (5/3)	3 (2/1)	
Recurrence of hepatitis C			
Presence/absence	12/9	7/7	0.68

Three patients who converted CSA to TAC were not included in this analysis.

## Discussion

The monitoring of graft function after LTx is a complex procedure. Evaluation of graft function commonly consists of liver blood biochemistry tests, liver biopsy, transient elastography, and hepatobiliary scintigraphy. Liver transplant patients receive either CSA or TAC as the main immunosuppressant. CSA has been shown to exert some anti-HCV properties *in vitro* [18] and is still frequently used in these patients. One animal study showed a negative effect of CSA on the excretion of the tracer ^99m^Tc-mebrofenin, thus possibly confusing the evaluation of liver function when comparing patients treated with CSA or TAC. The aim of this study was to evaluate whether CSA impaired the result of hepatobiliary scintigraphy using ^99m^Tc-mebrofenin or not. For that purpose, the results of HEF at 3 months and 1 year after LTx were compared between the CSA and TAC groups.

In recent years, CSA has not been used as the initial immunosuppressive drug at our institute, with the exception of HCV positive patients. To eliminate the recipients' factor of primary disease that might affect graft function, only patients who underwent LTx due to HCV cirrhosis were evaluated in this study.

There was no difference in recipient and donor age, recipient gender, malignancy rate, and CIT between the CSA and TAC groups, which indicates that there is no significant pre-LTx bias in the two patient groups. Neither was there any difference in the occurrence of acute rejection, which indicates that there was no significant difference of the immunosuppressive potency between the two groups.

The comparison of HEF between the groups showed significantly lower HEF values in the CSA group compared to the TAC group. In addition, none of the CSA patients had HEF of 100%, with the range being 11% to 90%, while in the TAC group, six patients (40%) acquired 100% of HEF (Tables 2 and 3).

In view of biochemical parameters, at 3-month follow-up, there was no significant difference of ALAT, ASAT, ALP, and GT between the two groups except bilirubin in the TAC group that was significantly lower than in the CSA group, although both were within normal range (Table 2). This difference may to some extent explain the difference in HEF between the two groups. However, at the 1-year follow-up, there was no significant difference in liver function tests, including bilirubin and renal function tests, between the two groups (Table 3). Moreover, the results of three patients converted from CSA to TAC (Table 4) showed that HEF value increased without any substantial difference of bilirubin in two cases (cases 1 and 2) and in spite of a drastically increased bilirubin in one case (case 3). The patients who developed post-operative bile duct complication in the CSA group remained with low HEF value in spite of a normalized bilirubin level after treatment.

Although a ‘cholestatic’ effect of CSA, in contrast to TAC, as indicated by a significantly higher level of serum bilirubin at 3 months post-operatively should be considered, we interpret these overall data, supported by the animal experimental study, as CSA causing an incorrect diagnosis of hepatic dysfunction with hepatobiliary scintigraphy through CSA interference not seen for TAC.

The evaluation of liver biopsy showed that the degree of inflammation was higher in the TAC group than in the CSA group. There was no difference in other histological parameters; fibrosis, steatosis, and incidence of hepatitis C recurrence. In spite of a higher degree of inflammation in the TAC-treated patients, the HEF values were significantly better than in the CSA group. The reason for the higher degree of inflammation in the TAC group is not clear. Moreover, the CSA-treated patients with normal liver function tests and normal histology presented with HEF values that only ranged from 17% to 79%.

Adenosine triphosphate (ATP)-binding cassette (ABC) subfamilies are bile canalicular transporters. ABC subfamily C member 2 (*Abcc2*) mediates the secretion of amphiphilic glutathione, glucuronide, and sulfate conjugates into bile. Excretion of ^99m^Tc-mebrofenin from hepatocytes is mainly dependent on *Abcc2*. CSA is known to be associated with the regulation of *Abcc2*-dependent canalicular transport of bile salts and drugs [17]. Thus, the administration of CSA can inhibit ^99m^Tc-mebrofenin excretion. An alternative mechanism is that low ^99m^Tc-mebrofenin excretion is due to an elevated serum bilirubin value [14]. If so, this could partly explain the HEF difference at 3 months but not at 1 year after LTx. Inflammation of hepatocytes normally inhibits the ^99m^Tc-mebrofenin excretion [19]. In our groups, this should have had a negative effect on TAC-treated patients. Presumably, such a possible effect was less important than the negative effect on HEF by CSA.

To our knowledge, this study is the only investigation in which a correlation between scintigraphy and several clinical liver transplantation parameters, including liver biopsy, that has been performed.

## Conclusions

Our finding has significant clinical implications as it indicates that the assessment of the liver graft function may be misleading, due to low HEF values in CSA-treated patients with otherwise good liver function. In conclusion, hepatobiliary scintigraphy in patients under CSA immunosuppression may show inadequately low HEF values.

## Abbreviations

ALAT: alanine aminotransferase
ALP: alkaline phosphatase
ASAT: aspartate aminotransferase
AZA: azathioprine
CIT: cold ischemia time
CSA: cyclosporin A
LTx: liver transplantation
HCV: hepatitis C virus
HEF: hepatic extraction fraction
GT: gamma-glutamyl transpeptidase
TAC: tacrolimus
SD: standard deviation
